# 14-3-3γ haploinsufficiency leads to altered dopamine pathway and Parkinson’s disease-like motor incoordination in mice

**DOI:** 10.1186/s13041-022-00990-z

**Published:** 2023-01-05

**Authors:** Eunsil Cho, Jinsil Park, Eun Mi Hwang, Hyung Wook Kim, Jae-Yong Park

**Affiliations:** 1 Department of Integrated Biomedical and Life Sciences, Korea University, Seoul, 02708 Korea; 2 BK21FOUR R&E Center for Learning Health Systems, Korea University, Seoul, 02841 Korea; 3 College of Life Sciences, Sejong University, Seoul, 05006 Korea; 4 Center for Functional Connectomics, Korea Institute of Science and Technology (KIST), Seoul, 02792 Korea; 5 ASTRION, Seoul, 02842 Korea

**Keywords:** 14-3-3γ, Dopamine, Motor incoordination, Parkinson’s disease

## Abstract

The 14-3-3 protein family with seven isoforms found in mammals is widely expressed in the brain and plays various roles in cellular processes. Several studies have reported that 14-3-3γ, one of the 14-3-3 protein isoforms, is associated with neurological and psychiatric disorders, but the role of 14-3-3γ in the pathophysiology of brain diseases is unclear. Although studies have been conducted on the relationship between 14-3-3γ protein and Parkinson’s disease (PD), a common neurodegenerative disorder with severe motor symptoms such as bradykinesia and rigidity, a direct connection remains to be elucidated. We recently showed that adult heterozygous 14-3-3γ knockout mice are hyperactive and exhibit anxiety-like behavior. In this study, we further characterized the molecular and behavioral changes in aged 14-3-3γ heterozygous mice to investigate the role of 14-3-3γ in the brain. We observed decreased dopamine levels and altered dopamine metabolism in the brains of these mice, including changes in the phosphorylation of proteins implicated in PD pathology. Furthermore, we confirmed that they displayed PD symptom-like behavioral deficits, such as impaired motor coordination and decreased ability to the nest-building activity. These findings suggest an association between 14-3-3γ dysfunction and PD pathophysiology.

**Supplementary Information:**

The online version contains supplementary material available at 10.1186/s13041-022-00990-z.

## Introduction

The 14-3-3 proteins were initially identified in bovine brain homogenates. They are widely expressed in the brain, accounting for approximately 1% of the total soluble proteins [1, 2]. The 14-3-3 family comprises seven isoforms, β, γ, ε, η, ζ, σ, and τ/θ, which have been reported in mammals [3–6]. The 14-3-3 proteins, which form homo- or hetero-dimers, bind to several partners and regulate their phosphorylation [3, 7–12]. The first identified function of these proteins is to modulate the degree of activation of tryptophan hydroxylase and tyrosine hydroxylase (TH) by regulating phosphorylation [13]. Through the interactions between the 14-3-3 proteins and their partners, these proteins play various roles in cellular processes such as cell cycle regulation, apoptosis, and intracellular signaling [14, 15].

Based on the abundance of 14-3-3 proteins in the brain, their functions in neurodegenerative and psychiatric diseases such as Creutzfeldt–Jakob disorder (CJD), Alzheimer’s disease (AD), Parkinson’s disease (PD), and schizophrenia have been studied [16, 17]. The 14-3-3 proteins are used as markers in the cerebrospinal fluid for CJD diagnosis, with high sensitivity and specificity [18, 19]. In the hippocampus of patients with AD, the 14-3-3 proteins co-localize with neurofibrillary tangles (NFT) [20]. Moreover, the expression of several 14-3-3 isoforms is increased in these patients [21]. These findings indicate that 14-3-3 proteins may play an important role in the development of NFT in AD.

An association between 14-3-3γ and PD has been suggested in several reports, including the first identified function of 14-3-3γ as an activator of TH [13]. PD is well-known as a common neurodegenerative disorder. A decrease in dopamine levels due to the loss of dopaminergic neurons in the substantia nigra is a known cause of PD [22]. It generally presents with both motor and non-motor symptoms and typically exhibits symptoms of motor defects, such as resting tremors, bradykinesia, rigidity, and postural instability [23]. Similar to AD, 14-3-3 proteins co-localize with the Lewy body (LB) by immunostaining [24, 25]. Additionally, the expression levels of 14-3-3γ and 14-3-3θ were significantly decreased in the α-synuclein (α-syn) overexpressed mice, an animal model of PD [26]. Moreover, researchers observed that overexpression of 14-3-3θ, 14-3-3ε, and 14-3-3γ reduces the toxicity in cells treated with 1-methyl-4-phenylpyridinium (MPP +) to induce PD-like cytotoxicity [26]. These findings suggest that the 14-3-3γ protein may be involved in PD pathology.

A previous report [27] showed several behavioral effects of 14-3-3γ deficiency in the brain using 14-3-3γ heterozygous knockout (HET) mice. The 14-3-3γ homozygous knockout mice we produced did not survive until postnatal day 21, which implies that 14-3-3γ plays a vital role in development. Moreover, the 14-3-3γ HET mice showed hyperactivity and depression-like behavior. Stress-sensitive behaviors were also observed in the 14-3-3γ HET mice compared to the littermate wild-type control mice (CTL). Based on the behavioral data, we suggest that reduced 14-3-3γ levels may be involved in neuropsychiatric diseases, such as attention deficit hyperactivity disorder (ADHD). Even though the phenotype of 14-3-3γ deficient mice has been partially identified, it is still insufficient to investigate the functions of 14-3-3γ. In this study, we demonstrate the effect of 14-3-3γ deficiency on dopamine processes and motor behaviors in aged 14-3-3γ HET mice.

## Materials and methods

### Animals

The 14-3-3γ deficient mouse was generated at Kumamoto University by random insertion of the gene trap vector pU-21 W into the exon 2 region of the *Ywhag* gene (Entrez Gene ID: 22628) located on the mouse chromosome 5qG2 [28]. In this study, we used the 14-3-3γ HET mice and CTL mice. The mouse card ID was 1462, and the strain name was B6;CB YwhagGt(pU-21W)266Car. The website address of the database for exchangeable gene trap clones is http://egtc.jp/action/access/clone_detail?id=21-W266. The mice were housed in polycarbonate cages under standard laboratory conditions (12-h light/dark cycle, 22 ± 2 °C, and 50 ± 5% humidity). Food and water were provided ad libitum. After performing all the behavioral tests, the mice were sacrificed using CO_2_ inhalation, and the brain tissue was collected. Collected samples were stored at 80 °C until analysis. All animal experiments followed the guidelines of the Institutional Animal Care and Use Committee of Sejong University and Korea University.

### Genotyping

For mouse genotyping using polymerase chain reaction (PCR), the following primer sequences were used: Knockout (KO) forward primer 5′-CTCAGGTGGTCTGATGGCAG-3′ and KO reverse primer 5′-TGGGGTTCTCCACATTGAGC-3′ for *Ywhag* knockout mice, and wild-type (WT) forward primer 5′-TCATCAGCAGCATCGAGCAG-3′ and WT reverse primer 5′-ATGGCGTCGTCGAAGGC-3′ for CTL mice. PCR products were detected using 2% agarose gel electrophoresis in tris acetate ethylene diamine tetra acetic acid (EDTA) buffer.

### Quantitative reverse transcription-polymerase chain reaction

Total RNA was extracted from the mouse brains using a Hybrid-RTM kit (Cat. No. 305-101, GeneAll, Seoul, Korea). The concentration and purity of RNA were measured at 260 nm and 280 nm, respectively, using Nanodrop 2000 spectrophotometer (RRID: SCR_018042, Thermo Scientific, Waltham, MA, USA). The complementary DNA was reverse transcribed from the RNA using the EcoDryTM premix cDNA synthesis kit (Cat. No. 639543, TAKARA, SHIGA, JAPAN). The primer sequences were as follows: Ywhag reverse transcriptase (RT) forward primer 5′-CCTACCGGGAGAAGATCGAG-3′ and Ywhag RT reverse primer 5′-TAGTTGTCCAGCAGGCTCAG-3′ for Ywhag (NM_018871) gene and glyceraldehyde 3-phosphate dehydrogenase (GAPDH) RT forward primer 5′-AATGTGTCCGTCGTGGATCT-3′ and GAPDH RT reverse primer 5′-AGACAACCTGGTCCTCAGTG-3′ for GAPDH (NM_001289726) gene. Reverse transcription-polymerase chain reaction (RT-PCR) was performed in 48-well plates using StepOne Real-Time PCR System (RRID: SCR_014281, Applied Biosystems, Waltham, MA, USA). Each sample was analyzed in triplicate. The expression levels of the target genes were normalized with GAPDH as an endogenous reference. The results were calculated using the 2^−ΔΔCt^ method [29].

### Western blotting

Brain tissues were homogenized in 4 mL RIPA buffer, 40 μL protease inhibitor cocktail, and 40 μL phosphate inhibitor cocktail and centrifuged at 4 °C at 13,000 rpm for 10 min. Protein concentrations were determined using the Bradford assay. Proteins were diluted to 3 mg/mL with 5X sample buffer and RIPA buffer and then boiled at 95 °C for 5 min. Proteins were separated using 10–15% sodium dodecyl sulfate–polyacrylamide gel electrophoresis and transferred to polyvinylidene difluoride membranes. The membranes were blocked with 5% skim milk in tris buffered saline buffer with Tween 20 for 50 min at 22 °C. They were then incubated overnight at 4 °C with anti-14-3-3γ (1:1000, Cat. No. sc-398423, Santa Cruz Biotechnology, Dallas, TX, USA), anti-Tyrosine Hydroxylase (TH) (1:1000, Cat. No. MAB318, Millipore, Burlington, MA, USA), anti- phosphorylated TH (S31) (1:1000, Cat. No. 12041, Cell Signaling Technology, Danvers, MA, USA), anti-Leucine-rich repeat kinase 2 (LRRK2) (1:500, Cat. No. ab133474, Abcam, Cambridge, UK), anti-phosphorylated LRRK2 (S910) (1:1000, Cat. No. ab133449, Abcam, Cambridge, UK), anti-phosphorylated LRRK2 (S935) (1:1000, Cat. No. ab133450, Abcam, Cambridge, UK), anti-dopamine transporter (DAT) (1:1000, Cat. No. DAT14-A, ALPHA DIAGNOSTIC, San Antonio, TX, USA), anti-glial fibrillary acidic protein (GFAP) (1:1000, Cat. No. 12389S, Cell Signaling Technology, Danvers, MA, USA), and anti-β actin (1:1000, Cat. No. A2066, Sigma-Aldrich, St. Louis, MO, USA). After incubation with the primary antibodies, the membranes were washed and incubated with horseradish peroxidase-conjugated secondary antibodies for 2 h. Protein bands were visualized using Fusion Solo software (RRID: SCR_016305, Vilber Lourmat, Collégien, France). Protein expressions were quantified using ImageJ software (RRID: SCR_003070, National Institutes of Health, Bethesda, MD, USA) and normalized by β-actin.

### Immunohistochemistry

For immunohistochemical staining, 40-week-old 14-3-3γ HET and CTL mice were used. Mouse brains were fixed with 4% paraformaldehyde dissolved in phosphate-buffered saline (PBS, pH 7.4) via intracardiac perfusion and dehydrated by immersing in 30% sucrose dissolved in PBS. Brain slices were obtained using CM1950 cryostat (RRID; SCR_018061, Leica Biosystems, Wetzlar, Germany). The brain sections were permeabilized in PBS containing 0.5% Triton X-100 for 45 min after antigen retrieval in Tris–EDTA (10 mM Tris, 1 mM EDTA, 0.02% Tween 20; pH 9.0) solution at 80 °C for 30 min. After washing, the brain sections were blocked with PBS containing 10% normal goat serum and 5% bovine serum albumin at 22 °C for 2 h. They were incubated overnight at 4 °C with the following primary antibodies: anti-YWHAG (1:200, Cat. No. HPA026918, ATLAS antibodies, Bromma, Sweden), anti-neuronal nuclei (NeuN) (1:1000, Cat. No. ab104224, Abcam, Cambridge, UK), anti-glial fibrillary acidic protein (GFAP) (1:500, Cat. No. PA1-10004, Invitrogen, Waltham, MA, USA), anti-Dopamine transporter (DAT) (1:500, Cat. No. DAT14-A, Alpha Diagnostic, San Antonio, TX, USA), anti-Tyrosine Hydroxylase (TH) (1:500, Cat. No. MAB318, Millipore, Burlington, MA, USA), anti-phosphorylated TH (S31) (1:500, Cat. No. 13041, Cell Signaling Technology, Danvers, MA, USA). The brain sections were washed and were incubated with appropriate Alexa Fluor 488-, 594-, or 647-conjugated secondary antibodies (1:400, Jackson ImmunoResearch, West Grove, PA, USA) for 2 h at 22 °C. The washed brain sections were further incubated with 4,6-diamidino-2-phenylindole staining solution for 15 min, placed on a microscope slide, and fixed with a mounting solution (Cat. No. H-1400, Vector Laboratories, Burlingame, CA, USA). Images were obtained using Nikon Eclipse Ti2 confocal microscope (RRID: SCR_021068, Nikon, Tokyo, JAPAN). Immuno-intensity was quantified using ImageJ software (RRID: SCR_003070, National Institutes of Health, Bethesda, MD, USA).

### Quantitative Dopamine measurement assay

Dopamine ELISA Kit (Cat. No. ab285238, K4219, Abcam, Cambridge, UK) was used for quantitative identification of dopamine in brain homogenate. The experimental procedure follows the protocol suggested in the kit. Briefly summarized as follows; Reconstitute the lyophilized dopamine standard with Standard/Sample dilution buffer to make serial dilutions. The proposed standard points of 100, 50, 25, 12.5, 6.25, 3.125, 1.56, and 0 ng/mL of dopamine solution were reacted simultaneously with the sample to draw the standard curve. Brains are extracted from mice, and the tissue is rinsed with ice-cold phosphate-buffered saline with a protease inhibitor to remove excess hemolytic blood. Homogenize the brain tissue using a glass homogenizer on ice. Centrifuge the brain homogenate at 5000 g for 5 min to recover the supernatant. Allow the reagents to be prepared immediately before use to room temperature. Wash the plates provided in the kit with 1X wash buffer. Add 50 µL of standards, samples, and controls into the appropriate wells. Immediately add 50 µL of Biotin-detection antibody working solution into each well. Cover with a plate sealer and gently pat to mix thoroughly, then incubate at 37 °C for 45 min. Discard the solution and wash it several times with 1 × wash solution. After completely removing the washing solution, add 0.1 mL of HRP-Streptavidin conjugate working solution into each well and incubate at 37 °C for 30 min. Discard the solution and wash it several times with 1X wash solution. After completely removing the wash solution, add 90 µL of TMB substrate into each well and incubate in the dark at 37 °C. After 15 min, add 50 µL of stop solution into each well. Read the results at 450 nm using a microplate reader Tecan infinite m200 pro (Tecan, Männedorf, Switzerland).

### Behavioral tests

#### Hindlimb clasping test

The hindlimb clasping test (HCT) was performed to assess motor coordination. The mice were suspended by their tail, and the behavior of the hindlimb was recorded on video for 15 s. The clasping score of the mice was estimated by at least two persons watching the videos. The standard score was based on a previous study [30]. Briefly, score 0 was given when both hindlimbs and toes were splayed; score 1, when one hindlimb was splayed and the other was not; score 2, when both hindlimbs were partially retracted; and score 3, when both hindlimbs moved completely close to the abdomen and the toes shrank.

#### Balance beam test

The balance beam test (BBT) was performed to assess motor coordination. The apparatus consisted of beams and a goal box. The wide beam was 12 mm wide and 100 cm long, while the narrow beam was 6 mm wide and 100 cm long. The start and finish lines were located 10 cm from the ends of the beams, and the distance between the lines was 80 cm. A black goal box (5 cm × 4.5 cm × 5.5 cm) with fist-sized bedding and two or three chows was perceived as a safe place rather than the beams for the mice. The beams were placed 50 cm above the floor. The fabric was installed 7.5 cm above the bottom to prevent injury when the mice fell from the beams. The goal box was placed on the opposite side of the start line. The mice were expected to cross the beams from the start line to the finish line with a maximum cut-off of 60 s. Tests were conducted for three trials for each beam with minimum 3-min intervals. When the mouse did not move on, the mouse’s rump was tapped to urge them to cross. Fifteen seconds after reaching the goal box, the mice were left to return to their cage. The tests were repeated until every mouse succeeded in three trials on the wide beam, and that day was considered a test day. Several mice failed to cross on the narrow beam, and the graphs were created, excluding the failed trials. The following cases were considered a failure: falling off the beam, failing to cross in 60 s, hanging upside down when crossing a beam, crawling with the abdomen attached to the beam, and returning to the start line.

#### Rota-rod test

A rota-rod test (RRT) was performed to assess basal motor activity. Before the training session began, the mice were placed for 60 s on a rotating rod (4 rpm, constant speed) for adaptation and were returned to their home cage for at least 5 min. These adaptation sessions were repeated until none of the mice had fallen. After achieving adaptation, the mice were placed on the rotating rod for 300 s (4–40 rpm, gradual accelerating speed) for training. They were subjected to four trials with at least 5-min intervals and returned to their home cage between the trials. After 24 h, the tests were conducted again. The test conditions were identical to those of the training sessions. The latency to fall off the rod was recorded with a maximum cut-off of 300 s. For example, if the mouse withstood the rotating rod for 300 s, the latency to fall was recorded as 300 s. The recorded times were then averaged.

#### U-shaped social interaction test

The U-shaped social interaction test (USIT) was used to assess social ability. An open-field apparatus was used for the USIT with modifications. The apparatus comprised a square arena (40 cm long) with 30 cm high walls, and a small wall (20 cm × 30 cm) was erected in the middle of one high wall. Thus, this small wall created two partitioned square arenas (20 × 20 cm) and a neutral section (40 × 20 cm). Of the two partitioned arenas, one was considered an interaction zone and the other an empty zone. The two partitioned arenas contained an empty “holding cell” (10.16 cm in diameter and 13.97 cm in height) at the corner during the habituation phase. Each mouse was placed in the neutral section and allowed to explore the entire apparatus for 5 min. After the habituation session, the mouse was returned to the cage, while a partner male mouse was placed under the holding cell (on a randomly selected side), and a triangle-shaped object was placed under the other holding cell. The interaction zone was identified as the arena with the partner mouse, and the empty zone was identified as the arena with the object. During the test phase, each mouse was placed in the neutral section and allowed to explore the entire apparatus for 5 min. The ANY-maze video tracking system recorded the time spent by the test mouse in investigating the novel/stranger mouse or object. The investigation was defined as the test mouse’s nose touching the holding cell or sniffing within 1 cm of the target.

#### Nestlet shredding test

The nestlet shredding test (NST) was performed to investigate nesting behavior indicative of PD. The animals were given one nestlet in their home cage weekly for acclimation. For the test, the mice were isolated in a single cage with clean bedding and received one nestlet, which was weighed beforehand. The other conditions were maintained similarly to those in their home cage. The next day, the nestlets were collected and weighed to assess the shredding. The mice were tested for two trials, and the data from the last trial was used for evaluation.

#### Nest building test

The nest building test (NBT) was performed to investigate nesting behaviors indicative of PD. The NST was considered as the training for NBT. The mice were subjected to NBT a day after the NST. The conditions for the NBT were the same as those for the NST, except for the bedding. For the accuracy of measurement, the bedding material used in the NBT was made with smaller particles than the daily bedding to prevent nesting using bedding and to induce nesting using only nestlets. After overnight incubation, each mouse was returned to its home cage. In the test cage, the height of the nest from the bottom to the top and the NBT score were measured. A standard score indicates how well the nest was built based on a previous study [31].

### Statistical analysis

GraphPad Prism 8.0.2 (GraphPad Software Inc., La Jolla, California, CA, USA) was used for statistical analysis. Levene’s test was used to determine whether the two groups had similar variances. Statistical significances were analyzed using a two-tailed unpaired student’s t-test or Welch’s t-test, followed by post hoc Tukey’s multi-comparison test. According to the tests, *P*-values < 0.05, < 0.01, or < 0.001 were considered statistically significant.

## Results

### 14-3-3γ is mainly expressed in neurons in the striatum and substantia nigra of the brain

**Fig. 1:**
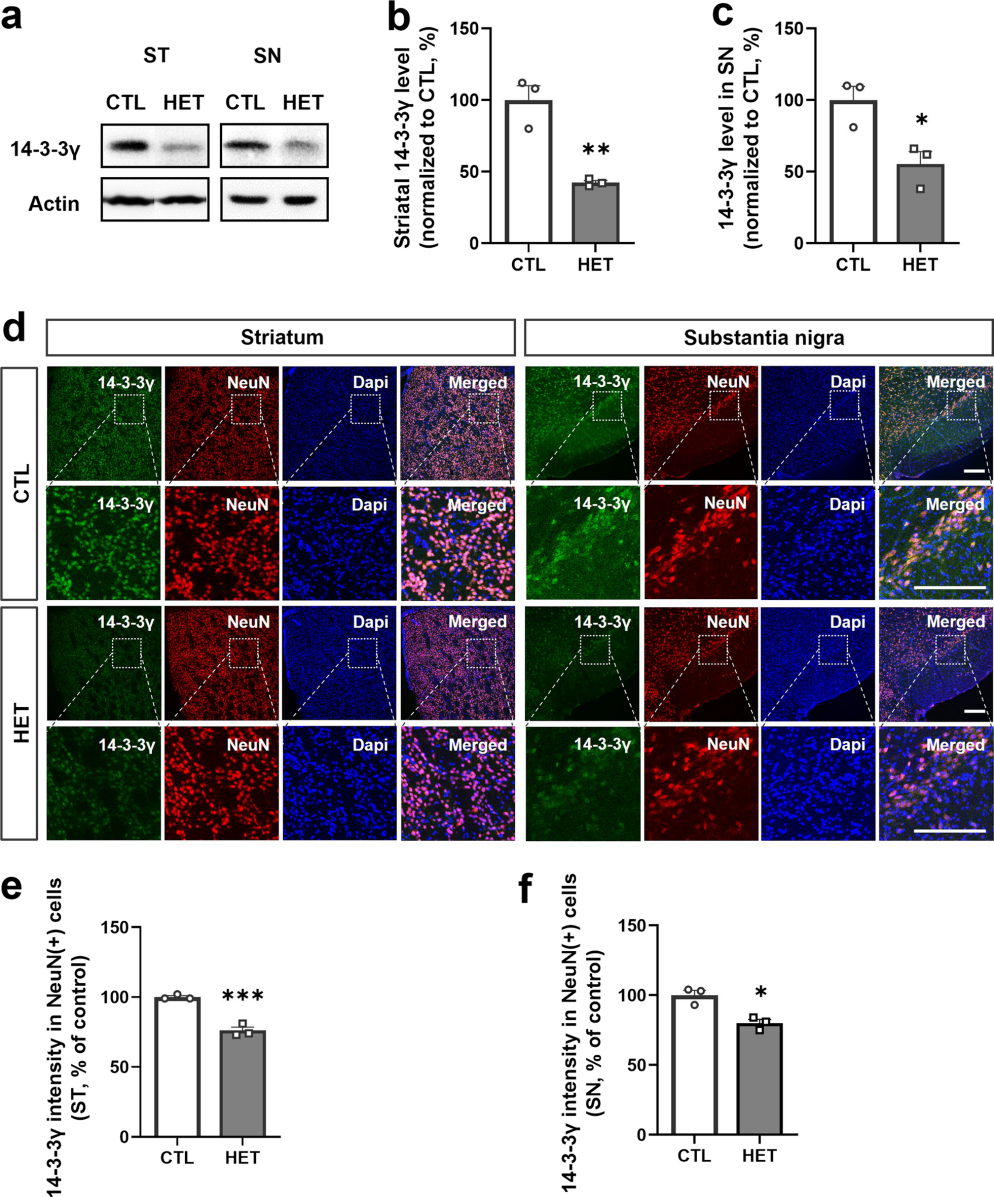
*Ywhag* knockout heterozygote mice identified with reduced expression of 14-3-3γ. **a–c** Representative western blotting image (**a**) and quantitative graph (**b,c**) show reduced expression of 14-3-3γ in the striatum (ST) and substantia nigra (SN) homogenates of the 14-3-3γ HET mice brain as compared to the CTL mice (*n* = 3 per group). **d** Immunohistochemistry results show that the 14-3-3γ immunostaining intensity in the ST and SN regions is mainly expressed in neurons indicated as NeuN ( +) cells, and a decrease in the 14-3-3γ intensity was detected in 14-3-3γ HET mice compared with CTL mice (scale bar: 0.2 μM). **e, f** Quantitative graphs of 14-3-3γ immunostaining intensity in NeuN ( +) cells in the ST (**e**) and the SN (**f**) regions of the 14-3-3γ HET mice brain and the CTL mice brain (*n* = 3 per group). Results are presented as means ± SEM; *P < 0.05, **P < 0.01 and ***P < 0.001. (*CTL* littermate wild-type control, *HET* heterozygous, *NeuN* neuronal nuclei, *DAPI* 4,6-diamidino-2-phenylindole, *SEM* standard error of mean)

The 14-3-3γ deficient mice were generated by random insertion of the gene trap pU-21 W vector into the exon 2 region of the *Ywhag* gene encoding 14-3-3γ located on the mouse chromosome 5qG2 (Additional file 1: Fig. S1a). The generation of knockout mice was identified by PCR-based genotyping using DNA obtained from the tail of mice on postnatal day 7 (Additional file 1: Fig. S1b). A previous report [27] showed that homozygous knockout mice died fatally. These findings indicate that HET 14-3-3γ knockout mice were expected to have developmental delays compared to the CTL mice. As abnormalities caused during development can lead to degenerative disorders and behavioral regression with age beyond developmental problems [32, 33], we investigated whether the 14-3-3γ levels in aged 14-3-3γ HET mice were still insufficiently maintained and whether there were changes in behavioral patterns in old age. Hence, the expression level of the *Ywhag* gene in the brains of 40-week-old aged 14-3-3γ HET mice was identified by quantitative RT-PCR (Additional file 1: Fig. S1c), and the expression level of 14-3-3γ protein was determined by western blotting (Additional file 1: Fig. S1d). The results confirmed that gene (Mean of HET, 54.79%) and protein expression level (Mean of HET, 39.67%) had been reduced by more than half in the aged 14-3-3γ HET mice (Fig. 1c and e).

Several studies have reported that 14-3-3γ is implicated in neurodegenerative disorders such as PD, AD, and CJD [16, 17]. Based on previously reported behavioral abnormalities in the 14-3-3γ HET mice [27], we focused on the striatum and substantia nigra regions of the brain involved in the dopaminergic pathway. Western blotting confirmed a significant reduction in 14-3-3γ expression in the striatum (Mean of HET, 42.33%) and the substantia nigra (Mean of HET, 55.33%) homogenates of 40-week-old 14-3-3γ HET mice (Fig. 1a–c). Moreover, immunohistochemistry showed that 14-3-3γ is predominantly co-localized with NeuN-positive cells, ensuring that it is mainly expressed in the neurons in these regions (Fig. 1d). It was also confirmed that the intensity of 14-3-3γ was significantly reduced in neurons described as NeuN-positive cells in the striatum (Mean of HET, 76%) and substantia nigra (Mean of HET, 80%) of 14-3-3γ HET mice brain (Fig. 1e and f).

### Reduced 14-3-3γ levels affect dopamine metabolism

**Fig. 2:**
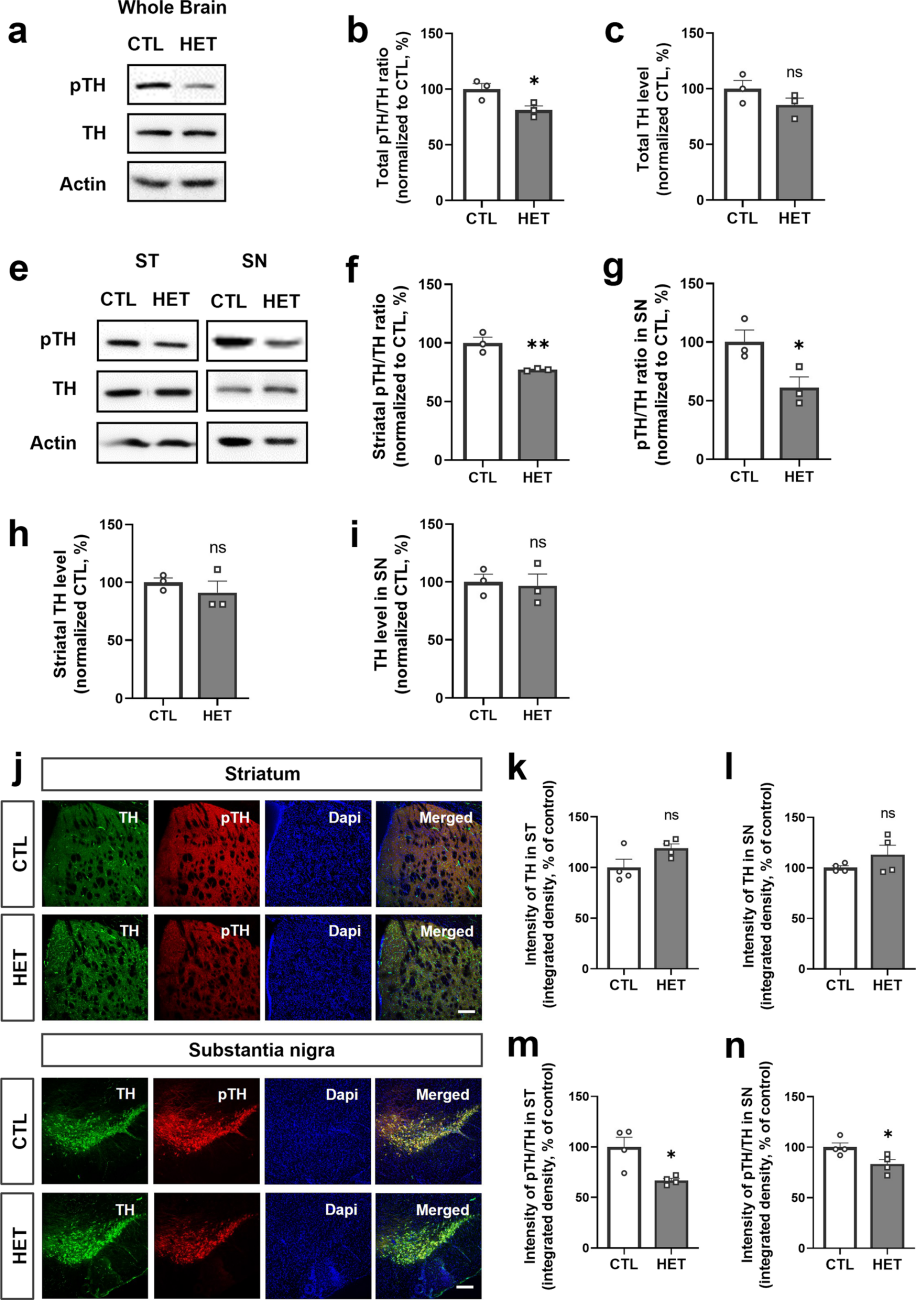
Decreased expression of phosphorylated tyrosine hydroxylase and insignificant change in expression of the tyrosine hydroxylase in the 14-3-3γ heterozygous mice brain. **a–c** Representative western blotting images (**a**) and quantitative graphs (**b, c**) of the phosphorylated tyrosine hydroxylase (pTH)/the tyrosine hydroxylase (TH) ratio (**b**) and total TH expression (**c**) in whole brain homogenates of the 14-3-3γ HET mice and CTL mice (*n* = 3 per group). **e-i** Representative western blotting images (**e**) and quantitative graphs (**f-i**) of pTH/TH ratio (**f,g**) and TH expression (**h,i**) in the striatum (ST) and the substantia nigra (SN) homogenates of the 14-3-3γ HET mice brain and CTL mice brain (*n* = 3 per group). **j** Immunohistochemistry results show reduced expression of pTH but not TH in the ST and the SN regions of the 14-3-3γ HET mice brain and CTL mice brain (scale bar, 0.2 μM). **k, l** Quantitative graphs of TH immunostaining intensity in the ST (**k**) and the SN (**l**) regions of the 14-3-3γ HET mice brain and the CTL mice brain (*n* = 4 per group). **m, n** Quantitative graphs of pTH immunostaining intensity normalized by TH in the ST (**m**) and the SN (**n**) regions of the 14-3-3γ HET mice brain and CTL mice brain (*n* = 4 per group). Results are presented as means ± SEM; *P < 0.05, **P < 0.01 and ***P < 0.001. (*CTL* littermate wild-type control, *HET* heterozygous, *pTH* phosphorylated tyrosine hydroxylase, *TH* tyrosine hydroxylase, *ST* striatum, *SN* substantia nigra, *NeuN* neuronal nuclei, *DAPI* 4,6-diamidino-2-phenylindole, *SEM* standard error of mean, *ns* non-significant)

To identify the association between 14-3-3γ and PD, we investigated the expression levels of proteins related to dopamine metabolism. Other researchers have reported that 14-3-3γ binds to tyrosine hydroxylase (TH), an enzyme required for the biosynthesis of catecholamines, including dopamine, and stabilizes and maintains the phosphorylation of TH [13, 34, 35]. Therefore, we investigated the expression levels of phosphorylated TH (pTH) and total TH using western blotting (Fig. 2a). In the whole brains of 14-3-3γ HET mice, there was no difference in overall TH levels (Fig. 2c), but pTH levels were significantly decreased (Mean of HET, 81.33%) (Fig. 2b). Furthermore, to investigate the degeneration of dopaminergic neurons and to determine whether there are differences in expression between PD-associated brain subregions, the levels of pTH and TH in the striatum and substantia nigra homogenates were confirmed using western blotting (Fig. 2e). As a result, a significant decrease in pTH was observed in both the striatum (Mean of HET, 77%) and substantia nigra (Mean of HET, 61%) of the 14-3-3γ HET mice brain, with a greater decrease in the substantia nigra than the striatum (Fig. 2f–i). In addition, there was no significant difference in the degree of TH intensity in the immunohistochemical reaction of the 14-3-3γ HET mice brain and CTL mice brain (Fig. 2j–i), whereas the intensity of pTH was significantly decreased in both the striatum (Mean of HET, 66.75%) and substantia nigra (Mean of HET, 83.25%) (Fig. 2m and n). These results suggest that the reduction in 14-3-3γ affects phosphorylation and activity of TH, although no significant degeneration of TH-expressing dopaminergic neurons was observed in the brain of 40-week-old 14-3-3γ HET mice.

**Fig. 3:**
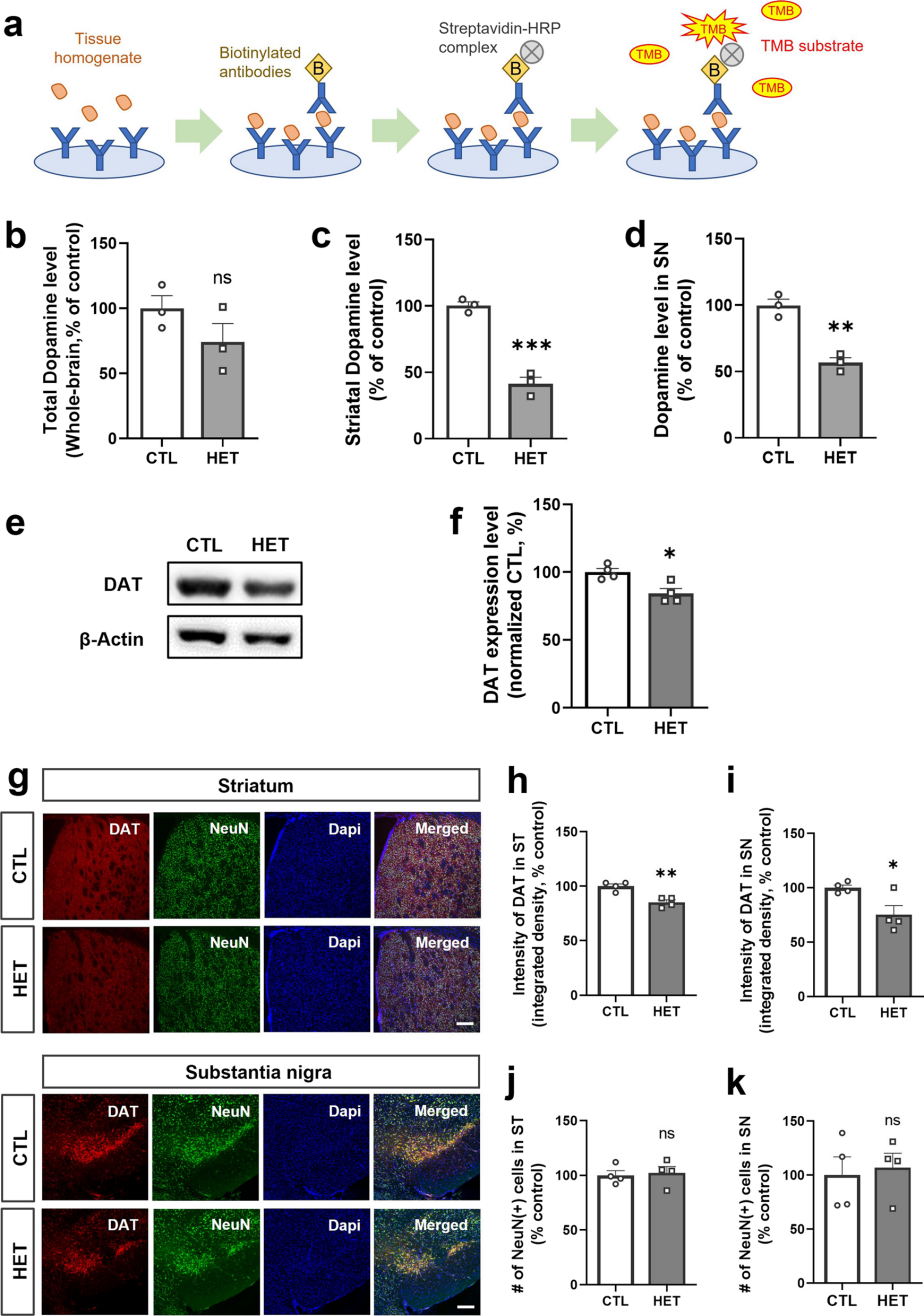
Decreased dopamine level and dopamine transporter expression in the 14-3-3γ heterozygous mice brain. **a** Schematic diagram of dopamine assay using competitive ELISA assay. **b–d** Results of dopamine levels in the whole brain (**b**), the striatum (ST) (**c**) and substantia nigra (SN) (**d**) homogenates of the 14-3-3γ HET mice brain and CTL mice brain (*n* = 3 per group). **e, f** Representative western blotting image (**e**) and quantitative graph (**f**) of the dopamine transporter (DAT) expression in brain homogenates of the 14-3-3γ HET mice and CTL mice (*n* = 4 per group). **g** Immunohistochemistry results showing reduced expression of DAT intensity in the ST and SN regions of the 14-3-3γ HET mice brain and CTL mice brain (scale bar, 0.2 μM). **h, i** Quantitative graphs of DAT immunostaining intensity in the ST (**h**) and SN (**i**) regions of the 14-3-3γ HET mice brain and CTL mice brain (*n* = 4 per group). Results are presented as means ± SEM; *P < 0.05, **P < 0.01 and ***P < 0.001. (*CTL* littermate wild-type control, *HET* heterozygous, *pTH* phosphorylated tyrosine hydroxylase, *TH* tyrosine hydroxylase, *DAT* dopamine transporter, *ST* striatum, *SN* substantia nigra, *NeuN* neuronal nuclei, *DAPI* 4,6-diamidino-2-phenylindole, *SEM* standard error of mean, *ns* non-significant)

Next, to determine whether the reduction of 14-3-3γ directly affects dopamine metabolic activity, as well as TH phosphorylation, dopamine levels in the brain of 14-3-3γ HET mice were measured by quantitative dopamine ELISA assay (Fig. 3a). As a result, the dopamine level of the whole brain homogenate of the 14-3-3γ HET mice was decreased compared to the CTL mice, but there was no significance (Fig. 3b). However, a significant decrease in dopamine levels was observed in the homogenates of the striatum (Mean of HET, 41.33%) and substantia nigra (Mean of HET, 56.67%) of 14-3-3γ HET mice (Fig. 3c and d).

Furthermore, significantly reduced expression of DAT, which maintains homeostasis by the reuptake of released dopamine into presynaptic neurons, was observed by western blotting in the 14-3-3γ HET mice brains (Mean of HET, 84.2%) (Fig. 3e and f). Similarly, immunohistochemistry showed a significant decrease in DAT expression in the striatum (Mean of HET, 85.25%) and substantia nigra (Mean of HET, 75%) (Fig. 3g–i). However, significant neuronal degeneration was not observed in the striatum and substantia nigra in the brain of 40-week-old 14-3-3γ HET mice since there was no change in the number of neurons expressed as NeuN (Fig. 3j and k). These results show that dopamine synthesis and reuptake processes are defect in the striatum and substantia nigra of aged 14-3-3γ HET mice.

### 14-3-3γ reciprocally affects the phosphorylation of leucine-rich repeat kinase 2

**Fig. 4:**
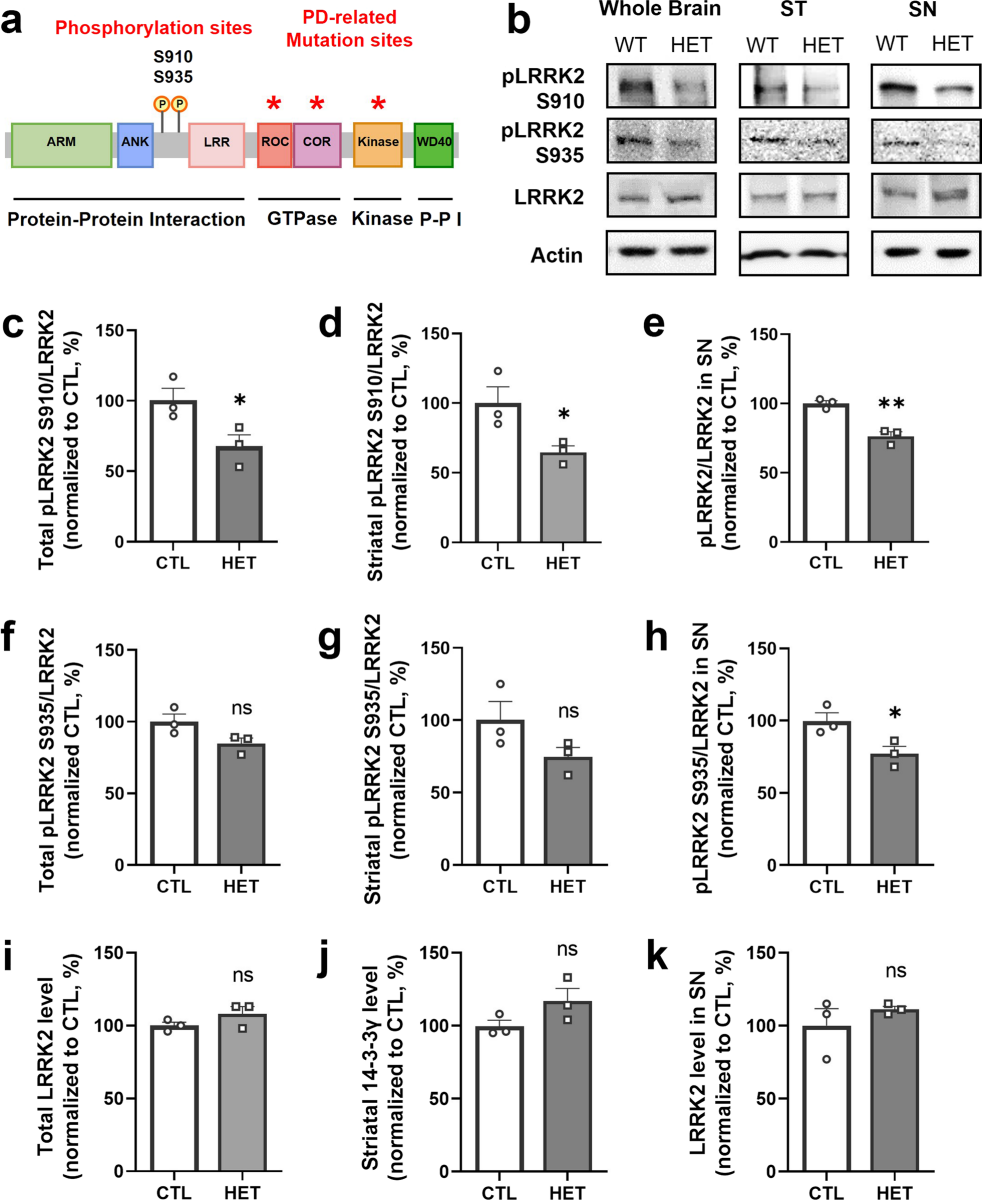
Decreased expression of the phosphorylated leucine-rich repeat kinase 2, but not total leucine-rich repeat kinase 2 expression in the 14-3-3γ heterozygous mice brain. **a** Schematic diagram of the domain structure of leucine-rich repeat kinase 2 (LRRK2) **b** Representative western blotting images of the pLRRK2 at S910, at S935, and LRRK2 expression in the whole brain, the striatum (ST), and substantia nigra (SN) homogenates of the 14-3-3γ HET mice and CTL mice. **c–k** Quantitative graphs of pLRRK S910 (**c**–**e**), S935 (**f–h**), and LRRK2 (**i–k**) expression in the whole brain, the ST, and SN homogenates of the 14-3-3γ HET mice and CTL mice (*n* = 3 per group). Results are presented as means ± SEM; *P < 0.05, **P < 0.01 and ***P < 0.001. (*CTL* littermate wild-type control, *HET* heterozygous, *P-P I* protein–protein interaction, *pLRRK* phosphorylated leucine-rich repeat kinase 2, *LRRK2* leucine-rich repeat kinase 2, *ST* striatum, *SN* substantia nigra, *SEM* standard error of mean, *ns* non-significant)

To address the association between 14-3-3 protein and PD, leucine-rich repeat kinase 2 (LRRK2) is one of the important factors to be investigated. Previous studies have identified 14-3-3 protein as a binding protein for LRRK2, a risk factor for PD [35–37]. Several genetic studies have found mutations in the GTPase or serine/threonine kinase domains of LRRK2 in both familiar and sporadic PD patients (Fig. 4a) [38, 39]. It has been suggested that LRRK2 mutations result in changes in biochemical activity and stabilization of the active state, resulting in abnormally excessive activity [40] and inappropriate interactions with binding partners in the protein–protein interaction domain (Fig. 4a) [41–44]. It is also associated with the clinical and pathological features of PD patients. Among the 14-3-3 proteins, 14-3-3γ is the isoform with the highest affinity for LRRK2 [36, 37]. Moreover, LRRK2 phosphorylation at Ser910 and Ser935 with high binding affinity to 14-3-3γ [45, 46] has been reported to be reduced in several mutant forms of LRRK2 associated with PD [47].

Therefore, we investigated the expression of total LRRK2 and phosphorylated LRRK2 at Ser910 and Ser935 in the whole brain, striatum, and substantia nigra homogenate of 14-3-3γ HET mice using western blotting (Fig. 4b). As a result, phosphorylated LRRK2 at Ser910 was significantly reduced in homogenates of the whole brain (Mean of HET, 67.67%) (Fig. 4c), the striatum (Mean of HET, 64.67%) (Fig. 4d), and substantia nigra of 14-3-3γ HET mice (Mean of HET, 76.33%) (Fig. 4e). In addition, phosphorylated LRRK2 at Ser935 was not respectably different between the 14-3-3γ HET mice and CTL mice in the whole brain and striatum homogenate (Fig. 4f and g), but a significantly decreased was observed in the substantia nigra of 14-3-3γ HET mice (Mean of HET, 77%) (Fig. 4h). However, there was no difference in overall LRRK2 expression in the brains of 14-3-3γ HET mice compared to the CTL mice, suggesting that reduction in 14-3-3γ does not affect total LRRK expression (Fig. 4i–k). Therefore, it is suggested that 14-3-3γ is implicated in the signaling mechanisms and activation of LRRK2 after the gene expression. These results indicate that not only LRRK2 phosphorylation at Ser910 and Ser935 is essential for phosphorylation-dependent binding of 14-3-3γ protein, but 14-3-3γ also affects phosphorylation of LRRK2 through mutual regulation in both directions, thereby regulating LRRK2 activity.

### 14-3-3γ heterozygous mice are implicated in astrogliosis

**Fig. 5:**
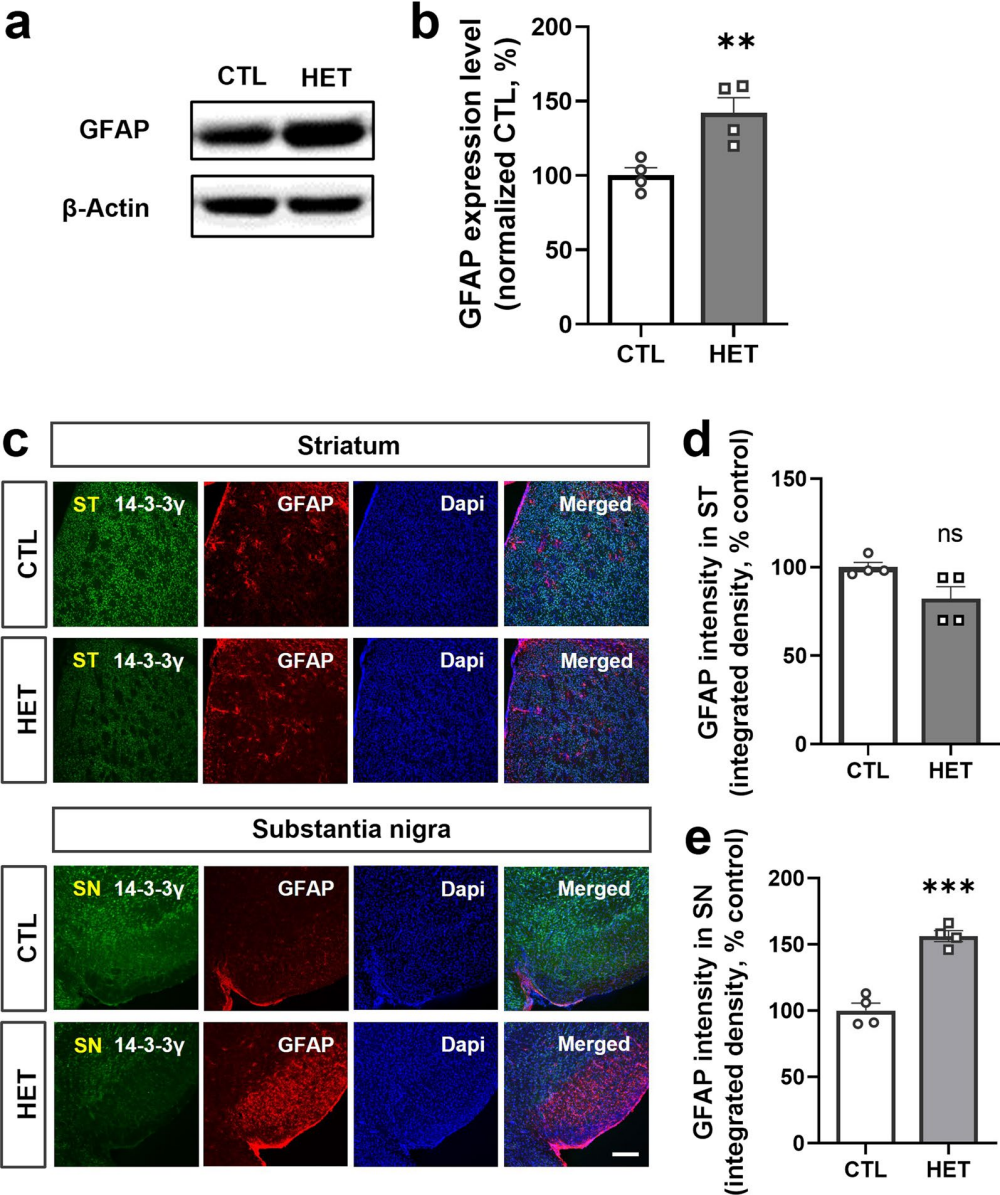
Increased expression of the glial fibrillary acidic protein in the substantia nigra of the 14-3-3γ heterozygous mice. **a, b** Representative western blotting image (**a**) and quantitative graph (**b**) of the glial fibrillary acidic protein (GFAP) in brain homogenates of the 14-3-3γ HET mice and CTL mice (*n* = 4 per group). **c** Immunochemistry results for GFAP expression enhanced in the substantia nigra (SN) region and not significantly enhanced in the striatum (ST) region in the 14-3-3γ HET mice brain and the CTL mice brain (scale bar, 0.2 μM). **d, e** Quantitative graphs of GFAP immunostaining intensity in the ST (**d**) and SN (**e**) regions of the 14-3-3γ HET mice brain and the CTL mice brain (*n* = 4 per group). Results are presented as means ± SEM; *P < 0.05, **P < 0.01 and ***P < 0.001. (*CTL* littermate wild-type control, *HET* heterozygous, *GFAP* glial fibrillary acidic protein, *ST* striatum, *SN* substantia nigra, *DAPI* 4,6-diamidino-2-phenylindole, *SEM* standard error of mean, *ns* non-significant)

Besides dopaminergic neurotransmission abnormalities and changes in phosphorylation and activity of related signaling proteins, the inflammatory response is also one of the major pathological hallmarks of PD. The glial fibrillary acidic protein (GFAP), a marker of activated astrocytes, is increased in patients and animal models of PD [48–51]. Therefore, we identified the expression level of GFAP in the brains of 14-3-3γ HET mice to determine whether a lack of 14-3-3γ is involved in astrogliosis similar to PD symptoms. An increase in the expression level of GFAP in the brain of 14-3-3γ HET mice compared with the CTL mice was observed in western blotting results (Fig. 5a). This increase was confirmed to be significant by quantification (Mean of HET, 142.3%) (Fig. 5b). In addition, immunohistochemistry was used to investigate the extent of GFAP expression in the striatum and substantia nigra of the 14-3-3γ HET mice and CTL mice (Fig. 5c). Our data show that GFAP levels are significantly increased, particularly in the substantia nigra of 14-3-3γ HET mice (Mean of HET, 156.3%) (Fig. 5d and e), indicating that a lack of 14-3-3γ may induce PD-like astrogliosis.

### 14-3-3γ heterozygous mice display defects in motor coordination

**Fig. 6:**
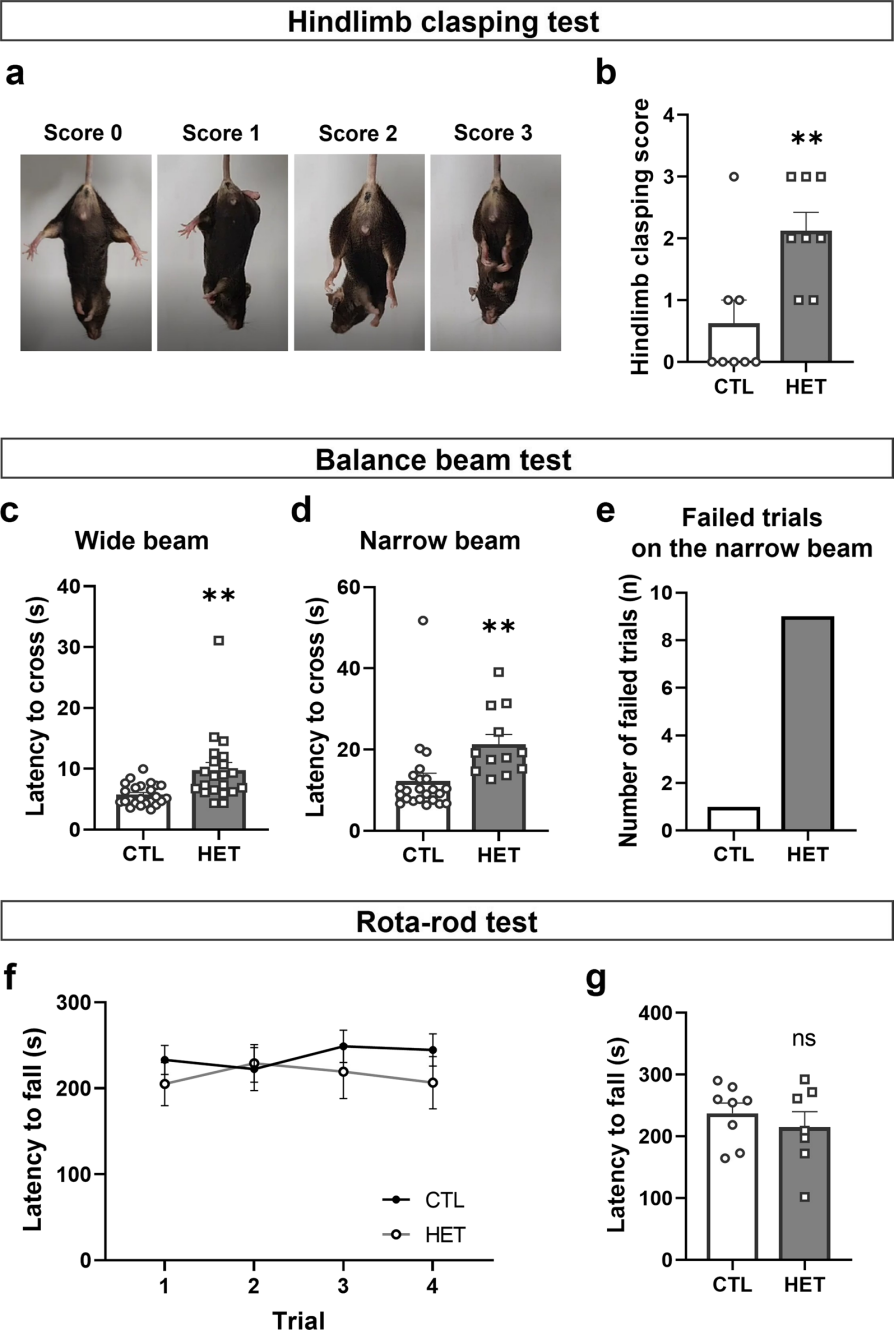
Defect in the motor coordination of 14-3-3γ heterozygous mice without a decrease in the basal motor activity. **a** Representative image showing the standard scoring system for the hindlimb clasping test (HCT). **b** The 14-3-3γ HET mice show a significant increase in the clasping scores (*n* = 8 per group). **c, d** Crossover latency in the 14-3-3γ HET mice was significantly increased for both wide and narrow beams in the balance beam test (BBT) (*n* = 24 CTL and *n* = 21 HET). **e** Images showing the failed trials of the 14-3-3γ HET mice on the narrow beam. **f** Fall latency was recorded for the 14-3-3γ HET and CTL mice on a rotating rod moving in an accelerated manner in four rota-rod tests (RRT) experiments (*n* = 8 CTL and *n* = 7 HET). **g** The mean of the data from the four trials presented here showed no significant difference in basal motor activity between groups. Results are presented as means ± SEM; *P < 0.05 and **P < 0.01. (*CTL* littermate wild-type control, *HET* heterozygous, *SEM* standard error of the mean)

Next, we performed several behavioral tests to determine whether the 14-3-3γ HET mice exhibited aberrant motor behavior typical of PD. We conducted the Hindlimb Clasping Test (HCT) to determine whether the 14-3-3γ HET mice have motor coordination problems since the hindlimb clasping has been generally observed in neurodegenerative diseases and can be used as a marker to assess disease progression stages [52–54]. Figure 6a shows representative images of the clasping scores ranging from 0 to 3 in our study. The 14-3-3γ HET mice displayed significantly higher clasping scores than the CTL mice (Mean of CTL, 0.6250; HET, 2.125) (Fig. 6b). The Balance Beam Test (BBT) was also conducted to confirm motor coordination ability [55], where the 14-3-3γ HET mice and CTL mice crossed a wide beam (12 mm) and a narrow beam (6 mm) during three times experiments. The latency time of the 14-3-3γ HET mice to cross increased for both wide (Mean of CTL, 5.753; HET, 9.798) and narrow beam (Mean of CTL, 12.23; HET, 21.32) (Fig. 6c and d). These results showed that the 14-3-3γ HET mice had more problems with balance and delicate motor control than the CTL mice. Both 14-3-3γ HET mice and CTL mice crossed the wide beam, but many 14-3-3γ HET mice failed to cross the narrow beam (CTL mice, one time, HET mice, nine times) (Fig. 6e). Thus, the 14-3-3γ HET mice failed to cross the narrow beam more and displayed a longer latency to cross the beams than the CTL mice.

Interestingly, the basal motor activity of 14-3-3γ HET mice was not altered. The Rota-Rod Test (RRT), which assesses the endurance of the mice on the accelerating rod, was performed to measure the basal motor activity. There was no statistically significant difference in the latency to fall between the 14-3-3γ HET mice and CTL mice in each trial (Fig. 6f) and the average of the four tests (Fig. 6f and g, respectively). These results suggest that reduced 14-3-3γ levels may lead to direct difficulties in motor coordination rather than decreased basal motor activity.

### 14-3-3γ heterozygous mice display defects in nest-building activity

**Fig. 7:**
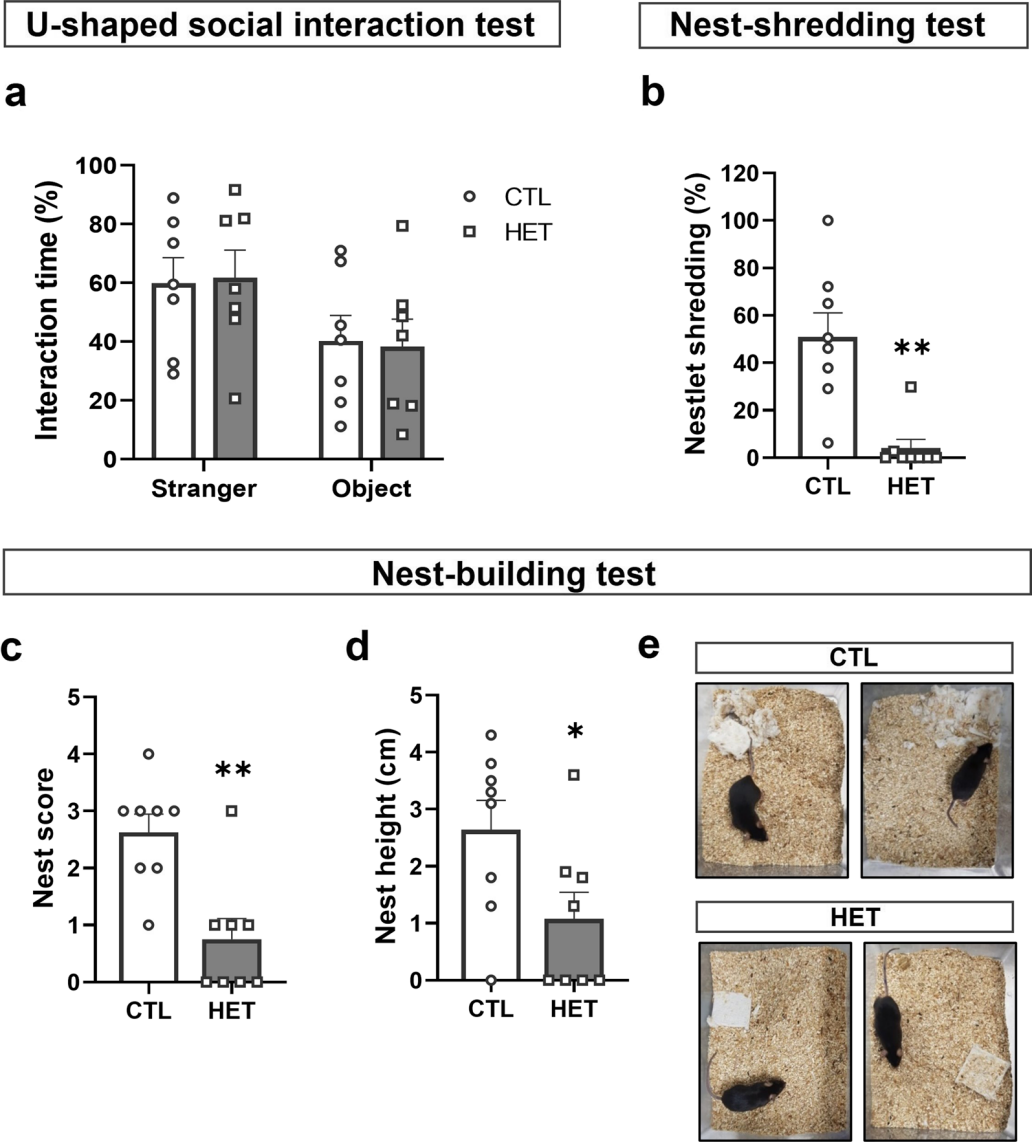
Nest building impairment without a social deficit in the 14-3-3γ heterozygous mice. **a** There was no significant difference in the interaction time (%) in the U-shaped social interaction test (USIT, interaction time [%] = $\frac{{\text{Time in the stranger mouse area}}}{{\text{Time in the triangle-shaped object }}{\text{me area}}}$ × 100, *n* = 7 per group). **b** The 14-3-3γ HET mice shred fewer nestlets than the CTL mice in the nest-shredding test (NST, nestlet shredding [%] = $\frac{{\text{Nestlet weight}}\,[\text{before}-\text{after}]}{{\text{Nestlet weight}}\,[\text{before}]}$ × 100, *n* = 8 per group). **c, d** The nest score (**c**) and nest height (**d**) were significantly decreased in the 14-3-3γ HET mice as compared to the CTL mice (*n* = 7 per group). **e** Representative pictures of the results for the 14-3-3γ HET mice and CTL mice in the nest-building test (NBT). Results are presented as means ± SEM; *P < 0.05 and **P < 0.01. (*HET* heterozygous, *CTL* littermate wild-type control, *SEM* standard error of the mean)

The U-shaped Social Interaction Test (USIT), a modified three-chamber social interaction test, was conducted in an open field box to assess social ability. Both the 14-3-3γ HET mice and CTL mice groups showed improvement in the rate of interaction time with the stranger mice but without a significant difference (Fig. 7a).

Following the USIT, the Nest-Shredding Test (NST) and the Nest Building Test (NBT) were conducted to confirm PD-related behaviors. The nesting behavior of rodents is sensitive to pathological disease states underlying changes in health or well-being. Therefore, it is known to have the potential as an early indicator of degenerative diseases, including PD. In this study, these tests were conducted in a single cage and scored individually. The USIT did not show the difference in social ability between the 14-3-3γ HET mice and CTL mice. On the other hand, the NST and NBT revealed significant differences in nesting-related behaviors between the 14-3-3γ HET mice and CTL mice. In the NST, the degree of nestlet shredding (%) in 14-3-3γ HET mice remarkably decreased (Mean of CTL; 50.89, HET; 4.075) (Fig. 7b). In the NBT, half of the 14-3-3γ HET mice did not build a nest at all, whereas the other half constructed nests of lower quality than the CTL mice with respect to the nest scores (Mean of CTL; 2.625, HET; 0.75) and nest heights (Mean of CTL; 2.638, HET; 1.075) (Fig. 7c–e). Consequently, the 14-3-3γ HET mice displayed PD-like behavior associated with nesting activity.

## Discussion

The 14-3-3 protein interacts with several partners and participates in various cellular processes. It regulates protein–protein or protein–DNA interactions and modulates the phosphorylation of their partner proteins [3, 8, 13]. As 14-3-3 proteins are abundant in the brain, they are associated with neurodegenerative diseases, including PD [16]. As a member of the 14-3-3 family, 14-3-3γ is also associated with PD [17]. It was detected in the LB of PD patients and was expressed at low levels in the α-syn overexpressed mice [24–26]. Moreover, 14-3-3γ showed protective effects in PD-induced cells [26]. Although several studies have suggested a correlation between 14-3-3γ and PD, the role of 14-3-3γ in PD remains unclear. In this study, we aimed to determine whether the 14-3-3γ reduction in vivo causes behavioral motor deficits and induces other molecular or metabolic changes associated with PD.

As a result, we found that reduced 14-3-3γ levels lead to a lower phosphorylated state of TH in the striatum and substantia nigra, but the total TH expression levels did not show any differences. These data suggest that 14-3-3γ is involved in regulating the phosphorylation levels of TH but not its expression levels. Our data corroborate the findings of other studies that the 14-3-3 proteins support the phosphorylation of TH and increase the activity and stability of TH [13, 34, 35]. As TH is a critical enzyme in the dopamine synthesis process, our results suggest that reduced 14-3-3γ levels might cause problems in thiamine synthesis. The 14-3-3γ HET mice also showed decreased dopamine levels and DAT expression levels in the brain. This could lead to insufficient dopamine reuptake and unstable dopamine homeostasis. Similar to our results, the 14-3-3ζ knockout mice showed abnormal motor behavior and decreased DAT [56], indicating that 14-3-3 proteins, including the γ and ζ forms, might be in regulating expression levels and motor activity. These data also suggest that reduced 14-3-3γ levels may induce dysfunctional dopamine metabolism.

Mutations and altered activity of LRRK2 are closely related to the pathophysiology of familial and sporadic PD [38, 39], and this is suggested to be involved in the location and degree of LRRK2 phosphorylation [35]. Previous studies have reported that all 14-3-3 proteins except 14-3-3σ can bind to LRRK2, but 14-3-3γ and -η show the highest affinity [36]. In addition, the binding of 14-3-3γ to LRRK2 has also been reported [45]. In our results, the decrease in LRRK2 phosphorylation observed in the brains of the 14-3-3γ HET mice suggests that simultaneous interactions between 14-3-3γ and LRRK2 may result in synergistic affinity values. In addition, our results show that the dissociation of 14-3-3γ from LRRK2 induces dephosphorylation at S910/S935 of LRRK2. This result suggests that reducing 14-3-3γ may increase the opportunity for other proteins to competitively bind to the site where LRRK2 binds to 14-3-3γ [45]. In addition, reduced binding of 14-3-3γ triggers the cytoplasmic accumulation of LRRK2, which may induce intracellular re-localization of LRRK2 [37].

Consequently, a decrease in 14-3-3γ could lead to an increase in the activity of LRRK2, similar to PD patients. Indeed, reduced 14-3-3 protein and LRRK2 interaction and increased LRRK2 kinase activity was observed in the brains of PD rodent models and postmortem PD patients [57]. Therefore, our results suggest that 14-3-3γ regulates the activity of LRRK2 by reciprocally interacting with the phosphorylation of the binding sites, thereby contributing to the pathological characteristics of PD. Conversely, the binding of 14-3-3γ may induce structural or chemical changes in LRRK2, converting the LRRK2 kinase to an inactive form. For 14-3-3γ to modulate the activity of LRRK2 and become a new strategy to treat Parkinson's disease, a better understanding of the functional network of 14-3-3γ and further research into the molecular processes of its interactions with key partners is required.

The 14-3-3γ HET mice also exhibit motor control problems. The HCT showed retraction of the hindlimbs and shrinking of the toes in the 14-3-3γ HET mice in our study. This is indicated by the clasping scores, and the 14-3-3γ HET mice showed a higher clasping score than the CTL mice. In the BBT, the 14-3-3γ HET mice displayed defects in motor control, such as a long time to cross the beams and more failed trials compared to the CTL mice. However, they did not show decreased basal motor function during RRT. These behavioral characteristics in the 14-3-3γ HET mice seem to mimic bradykinesia and rigidity, among the motor symptoms of PD. These data indicate that the 14-3-3γ HET mice have defects in the motor coordination ability but not in the basal motor activity and strength. It is also expected that the behavioral abnormalities caused by the decrease of 14-3-3γ in 40-week-old heterogeneous mice will show a pattern close to the initial PD symptoms. Since the reduction in the 14-3-3γ level leads to motor coordination deficiency, one possibility is that dopamine synthesis or reuptake by the nerve terminals in the striatum is decreased due to reduced 14-3-3γ levels.

Additionally, 14-3-3γ deficiency leads to degraded behavioral activity in nest building. Building nests is extremely important in nature for protection, maintaining body temperature, and reproduction. Deficiencies in nesting activities represent diminished well-being or decreased social ability [58, 59]. However, in addition to motor impairment behaviors, some researchers have found that the PD model mice do not build nests [60–62]. Although the detailed mechanism remains unknown, researchers have suggested that reduced dopamine impairs reward circuitry and motor control ability [62, 63]. These changes may make the patients less likely to feel the need to build a nest or may reduce their ability to build nests. Making a nest is a known dopamine-dependent behavior and requires delicate forelimb movement [60–62]. The 14-3-3γ HET mice showed decreased nestlet-shredding behavior and deficient nest-building activity. In the USIT, both the 14-3-3γ HET and CTL mice showed normal social abilities, which indicates that the impaired nest-building activity is not related to social interaction problems but is caused by defects in the dopamine process.

GFAP, a marker of activated astrocytes, is generally highly expressed in injured brain regions. In postmortem brain tissues of patients with PD, enhanced GFAP expression has been observed in the substantia nigra, frontal cortex, and caudate nucleus [48–50]. In other studies, animal PD models, such as 6-hydroxydopamine or MPP + treated rats, displayed increased GFAP levels in the substantia nigra [50, 51]. We found that the 14-3-3γ HET mice showed remarkably increased GFAP protein levels in the substantia nigra. Our data are consistent with data on astrogliosis in PD patients and animal models of PD. These results suggest that reduced 14-3-3γ levels activated the astrocytes and damaged them, leading to astrogliosis in the brains of 14-3-3γ HET mice. However, other researchers presented no correlation between PD and activated astrocytes [64, 65]. Therefore, further research is needed regarding the association between 14-3-3γ, astrogliosis, and PD.

Our previous report found that the 14-3-3γ HET mice exhibited hyperactivity and depression-like and stress-sensitive behaviors compared to the CTL mice [27]. Previously, we suggested that 14-3-3γ might be involved in neuropsychiatric diseases and that the 14-3-3γ HET mice could be a model for ADHD. In this study, we also found a relation between 14-3-3γ and PD. We demonstrated molecular alterations related to dopamine processes and astrogliosis in mice with 14-3-3γ haploinsufficiency. They also exhibited PD-like behaviors, such as motor incoordination and defects in nest-building activity. Thus, our data provide evidence that reduced 14-3-3γ levels contribute to the impairment of dopamine metabolism and motor behavior deficits associated with PD.

## Conclusion

Our results indicate that reduced 14-3-3γ levels in vivo cause PD-related molecular changes and behaviors. These findings provide insight into the relationship between 14-3-3γ and PD. Thus, the 14-3-3γ HET mice may be a valuable model for studying the role of 14-3-3γ in PD. However, further studies are required to determine the direct effect of 14-3-3γ deficiency on PD, such as assessing whether PD drugs (e.g., levodopa) are effective in recovering the motor symptoms in the 14-3-3γ HET mice.

## Supplementary Information

**Additional file 1: Figure S1.** Generation strategy and validation of *Ywhag* knockout mice. **a** Schematic diagram of the gene targeting strategy for generating *Ywhag* knockout (14-3-3γ KO) mice. Gene trap vector pU-21 W was inserted into the exon 2 region of *Ywhag* located in the mouse chromosome 5qG2. **b** Genotyping result of the 14-3-3γ HET mice and the CTL mice. The 14-3-3γ KO mice are prenatally lethal. **c** Comparison of *Ywhag* mRNA expression by qRT-PCR in the brains of the 14-3-3γ HET mice and the CTL mice (*n* = 3 CTL and *n* = 5 HET). **d,e** Representative western blotting image (**d**) and quantitative graph (**e**) of 14-3-3γ protein expression in brain homogenates of the 14-3-3γ HET mice and the CTL mice (*n* = 3 per group). Results are presented as means ± SEM; *P < 0.05 and **P < 0.01. (CTL, littermate wild-type control; HET, heterozygous; qRT-PCR, quantitative reverse transcription-polymerase chain reaction; SEM, standard error of mean)

## Data Availability

The datasets generated during the current study are available from the corresponding authors upon reasonable request.
