# Supplementary figures and images for: 14-3-3γ haploinsufficiency leads to altered dopamine pathway and Parkinson’s disease-like motor incoordination in mice
Source: Mol Brain. 2023 Jan 5;16:2. doi: 10.1186/s13041-022-00990-z (PMC9817279; doi:10.1186/s13041-022-00990-z)

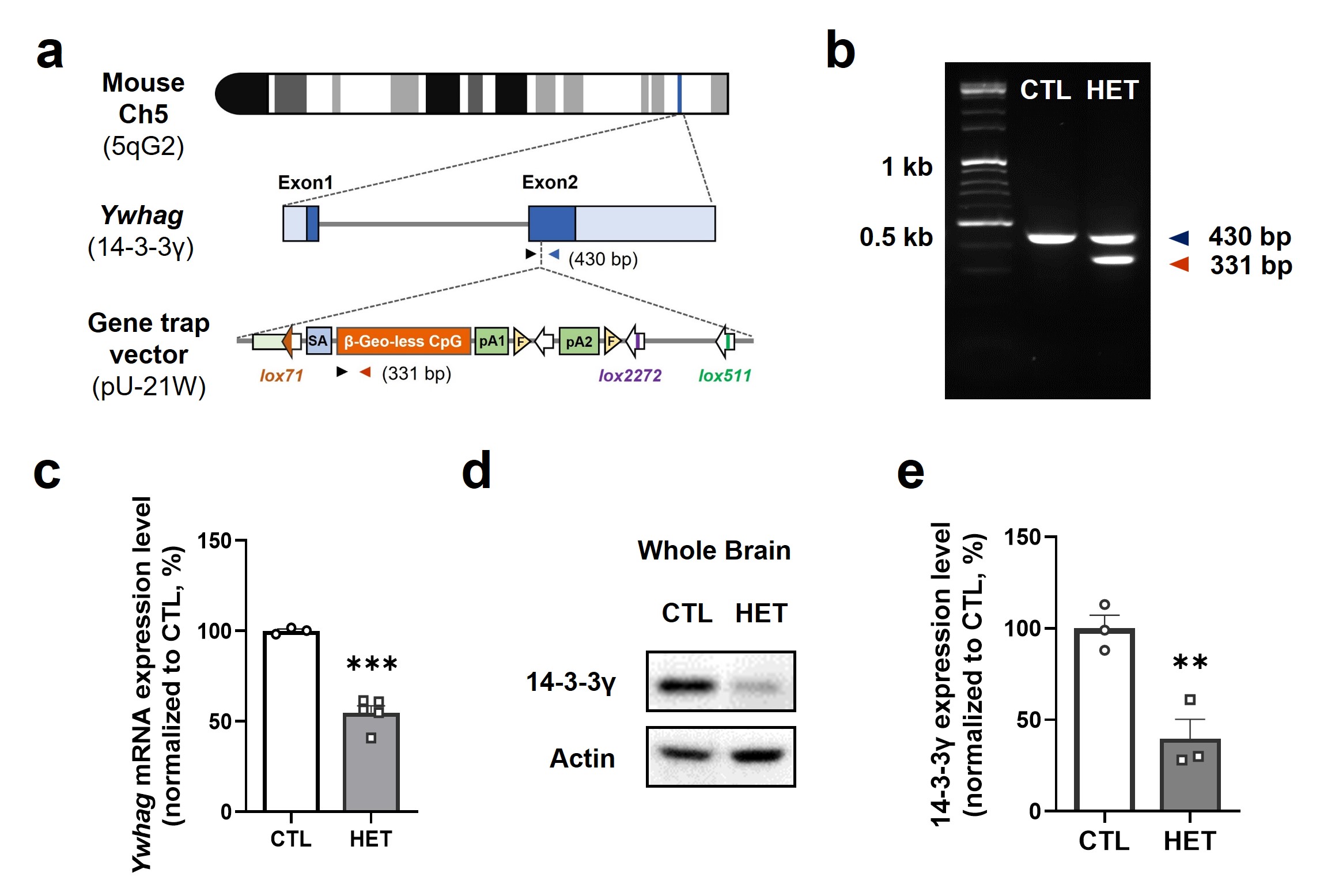

Supplement: Supplementary file 1 — Additional file 1: Figure S1. Generation strategy and validation of Ywhag knockout mice. a Schematic diagram of the gene targeting strategy for generating Ywhag knockout (14-3-3γ KO) mice. Gene trap vector pU-21 W was inserted into the exon 2 region of Ywhag located in the mouse chromosome 5qG2. b Genotyping result of the 14-3-3γ HET mice and the CTL mice. The 14-3-3γ KO mice are prenatally lethal. c Comparison of Ywhag mRNA expression by qRT-PCR in the brains of the 14-3-3γ HET mice and the CTL mice (n = 3 CTL and n = 5 HET). d,e Representative western blotting image (d) and quantitative graph (e) of 14-3-3γ protein expression in brain homogenates of the 14-3-3γ HET mice and the CTL mice (n = 3 per group). Results are presented as means ± SEM; *P < 0.05 and **P < 0.01. (CTL, littermate wild-type control; HET, heterozygous; qRT-PCR, quantitative reverse transcription-polymerase chain reaction; SEM, standard error of mean) [file 13041_2022_990_MOESM1_ESM.jpg]
